# Anxiety in Hospitalized Patients With Mild COVID-19 Before and After Psychological Intervention: A Retrospective Study

**DOI:** 10.7759/cureus.101575

**Published:** 2026-01-14

**Authors:** Gajendra Singh Lodwal, Govind Singh, GD Koolwal

**Affiliations:** 1 Physical Medicine and Rehabilitation, Dr. Sampurnanand Medical College, Jodhpur, IND; 2 Psychiatry, Vyas Medical College, Jodhpur, IND

**Keywords:** clinical anxiety, covid 19, hamilton anxiety rating scale (ham-a), hospitalized patient, psychological intervention

## Abstract

Background and objective

Hospitalization for coronavirus disease 2019 (COVID-19) is frequently associated with psychological distress. Anxiety can develop due to isolation, fear of disease progression, uncertainty regarding recovery, and disruption of normal social support networks. Early recognition of anxiety and implementation of structured psychological interventions during hospitalization may help reduce distress and improve patient well-being. This study aimed to evaluate the severity of anxiety at hospital admission and after seven days of structured psychological intervention among patients hospitalized with mild COVID-19.

Methods

This retrospective observational study included 100 patients hospitalized with laboratory-confirmed mild COVID-19. Anxiety severity was assessed using the Hamilton Anxiety Rating Scale (HAM-A) at admission (Day 0) and after seven days of psychological intervention (Day 7). Anxiety was categorized as mild (≤16), moderate (17-24), or severe (≥25). Changes in anxiety scores were analyzed, and associations between baseline anxiety severity and age, sex, and comorbidity status were assessed using the chi-square test.

Results

At admission, 36 (36%) patients had mild anxiety, 36 (36%) had moderate anxiety, and 28 (28%) had severe anxiety. The mean HAM-A score was 19.78 ± 10.94. After seven days of psychological intervention, 90 (90%) patients had mild anxiety and 10 (10%) had moderate anxiety, with complete resolution of severe anxiety. The mean HAM-A score decreased to 8.01 ± 5.62, representing a mean reduction of 11.77 points (59.5%). No statistically significant association was observed between baseline anxiety severity and age, sex, or comorbidity status (p>0.05).

Conclusions

Anxiety is common among patients hospitalized with mild COVID-19. In our cohort, structured psychological intervention during hospitalization was associated with significant short-term improvement in anxiety severity.

## Introduction

The coronavirus disease 2019 (COVID-19) pandemic has significantly stressed healthcare systems worldwide and has been associated with widespread adverse effects on mental health [1-3]. In addition to physical illness, patients hospitalized with COVID-19 frequently experience psychological distress resulting from isolation, concerns about disease progression, uncertainty regarding recovery, and reduced contact with family members. Hospital-related anxiety has been linked to poor coping mechanisms, sleep disruption, decreased adherence to treatment, and slower recovery [4]. Research conducted during the COVID-19 pandemic has consistently demonstrated a high burden of anxiety among hospitalized patients, including those with mild illness not requiring intensive respiratory support [5-7]. Despite this, evidence on short-term changes in anxiety following structured psychological interventions among hospitalized patients with mild COVID-19 remains limited.

The Hamilton Anxiety Rating Scale (HAM-A) is a widely used clinician-administered instrument designed to assess both psychic and somatic components of anxiety and is sensitive to short-term clinical change [8,9]. This study aimed to assess anxiety severity at admission and after seven days of structured psychological intervention in patients hospitalized with mild COVID-19 and to examine associations between baseline anxiety severity and selected demographic and clinical variables.

## Materials and methods

Study design and setting

This retrospective observational study was conducted at Dr. Sampurnanand Medical College, Jodhpur, and its affiliated COVID-19-designated hospitals. Medical records from September 2020 to September 2021 were reviewed. All patients hospitalized with laboratory-confirmed mild COVID-19 during the study period who met the inclusion criteria and had complete anxiety assessments were included, and no random sampling was performed. Mild disease was defined as symptomatic infection with oxygen saturation ≥94% on room air, without the requirement for supplemental oxygen or ICU admission. A total of 100 eligible patients meeting the inclusion criteria were selected from hospital records.

Inclusion and exclusion criteria

Patients aged 21 years or older with documented anxiety assessments at admission and at seven days were included. Patients with incomplete medical records, pre-existing major psychiatric disorders, or cognitive impairment interfering with reliable anxiety assessment were excluded.

Data collection

Extracted data included age, sex, presence of comorbid medical conditions, HAM-A scores, and anxiety categories at admission and Day 7, and details of psychological intervention.

Anxiety assessment

Anxiety severity was assessed in all included patients at admission using the Hamilton Anxiety Rating Scale, which quantifies the severity of anxiety symptoms. The scale was originally developed by Hamilton in 1959 and is widely used in clinical settings [8]. Anxiety severity was categorized as mild (≤16), moderate (17-24), or severe (≥25).

Psychological intervention

All patients received structured, non-pharmacological psychological intervention during hospitalization. The intervention included psychoeducation regarding anxiety symptoms, reassurance, supportive counseling, relaxation and breathing exercises, basic cognitive-behavioral techniques, stretching exercises, and guided meditation. Sessions were conducted by a psychologist under the supervision of a psychiatrist, with a minimum of three sessions, each lasting 30-40 minutes, delivered over seven days [10,11].

Ethical approval

The study was approved by the Institutional Ethics Committee, Dr. SNMC, Jodhpur. Informed consent was waived due to the retrospective design of the study. Patient confidentiality was maintained in accordance with the Declaration of Helsinki.

Statistical analysis

Categorical variables were expressed as frequencies and percentages, and continuous variables as mean ± standard deviation (SD) and median. Changes in anxiety scores were calculated. Changes in mean HAM-A scores between Day 0 and Day 7 were analyzed using a paired t-test. Associations between baseline anxiety severity and age, sex, and comorbidity status were analyzed using the chi-square test. A p-value <0.05 was considered statistically significant. Statistical analysis was performed using SPSS Statistics software version 26 (IBM Corp., Armonk, NY).

## Results

Study population and baseline characteristics

A total of 100 patients hospitalized with mild COVID-19 were included in the final analysis. The mean age of the study population was 48.31 years. Of the total patients, 66 (66%) were male, and 34 (34%) were female. Comorbid medical conditions were present in 40 (40%) patients, while 60 (60%) patients had no documented comorbidities. The demographic and clinical characteristics of the study population are summarized in Table 1.

**Table 1:** Demographic and clinical characteristics of the study population (N = 100) COVID-19: coronavirus disease 2019

Variable	Values
Mean age (years)	48.31
Male, n (%)	66 (66%)
Female, n (%)	34 (34%)
Comorbidity present, n (%)	40 (40%)
No comorbidity, n (%)	60 (60%)
Mild COVID-19, n (%)	100 (100%)

Anxiety severity

Anxiety Severity at Admission (Day 0)

At the time of hospital admission, all patients exhibited symptoms of anxiety as assessed using the Hamilton Anxiety Rating Scale. Based on anxiety severity categories, 36 (36%) patients had mild anxiety, 36 (36%) had moderate anxiety, and 28 (28%) had severe anxiety. The mean Hamilton Anxiety Rating Scale score at admission was 19.78 ± 10.94, with a median score of 19.

Anxiety Severity After Psychological Intervention (Day 7)

Following seven days of structured psychological intervention during hospitalization, a marked improvement in anxiety severity was observed. At the Day 7 assessment, 90 (90%) patients were categorized as having mild anxiety, while 10 (10%) patients continued to have moderate anxiety. No patients remained in the severe anxiety category after the intervention period. The mean Hamilton Anxiety Rating Scale score at Day 7 decreased to 8.01 ± 5.62, with a median score of 8.

Change in Anxiety Severity Over Time

The mean HAM-A score decreased by 11.77 points, corresponding to a 59.5% reduction from baseline. There was a complete resolution of severe anxiety. Comparison of HAM-A scores between admission and Day 7 using a paired t-test demonstrated a statistically significant reduction in anxiety severity (mean difference = 11.77, p < 0.001). These changes are detailed in Table 2.

**Table 2:** Anxiety severity categories and change in HAM-A scores at admission and Day 7 (N = 100) HAM-A: Hamilton Anxiety Rating Scale; SD: standard deviation

Parameter	Day 0	Day 7	Change
Anxiety severity category			
Mild (≤16), n (%)	36 (36%)	90 (90%)	+54
Moderate (17–24), n (%)	36 (36%)	10 (10%)	−26
Severe (≥25), n (%)	28 (28%)	0 (0%)	−28
HAM-A score			
Mean ± SD	19.78 ± 10.94	8.01 ± 5.62	−11.77
Median	19	8	−11
Percentage reduction in mean score	—	—	59.5%

Association between anxiety severity and demographic Variables

Analysis of baseline anxiety severity in relation to demographic and clinical variables showed no statistically significant association between anxiety severity and age, sex, or comorbidity status. Anxiety symptoms were observed across all demographic subgroups.

## Discussion

The findings of this study indicate that anxiety is common among patients hospitalized with mild COVID-19, with many individuals presenting with moderate to severe symptoms at admission. These observations are consistent with earlier reports describing a considerable psychological burden among hospitalized patients during the pandemic, including those without severe clinical disease [5-7]. A key observation of this study is the marked reduction in anxiety severity following structured psychological intervention during hospitalization. The nearly 60% reduction in mean HAM-A scores within seven days, as shown in Table 2, is comparable to outcomes reported in studies evaluating early psychological and psychosocial support among hospitalized patients with coronavirus disease 2019 [10,11]. These findings support the integration of structured psychological care into routine inpatient management.

Although a significant reduction in anxiety severity was observed following structured psychological intervention, these findings should be interpreted cautiously. The retrospective design and lack of a control group mean improvement cannot be attributed solely to the intervention. Natural symptom resolution over time, reassurance associated with hospitalization, medical stabilization, and adaptation to the hospital environment may also have contributed to the reduction in anxiety scores. Thus, causality cannot be firmly established, and results indicate association rather than direct effect. Similar improvements in anxiety and psychological distress have been noted in hospitalized COVID-19 patients receiving early supportive counseling, psychoeducation, and relaxation-based interventions, suggesting that brief non-pharmacological approaches can be beneficial in inpatient care [10-12]. The magnitude of improvement here aligns with prior reports, supporting the clinical relevance of structured psychological interventions in mild disease.

The absence of a significant association between baseline anxiety severity and demographic variables such as age and sex aligns with reports indicating that psychological distress during the COVID-19 pandemic affects hospitalized patients across demographic subgroups [6,13]. Similarly, the lack of association with comorbidity status suggests that hospitalization-related psychosocial stressors may contribute more substantially to anxiety than underlying medical conditions alone. This observation highlights the role of situational factors such as isolation, uncertainty regarding disease progression, and fear of transmission to family members as potential contributors to anxiety, irrespective of baseline medical risk profiles.

Although most patients demonstrated substantial improvement, a small proportion continued to experience moderate anxiety at Day 7. This observation is consistent with longitudinal studies reporting persistent anxiety symptoms among some survivors beyond the acute phase of COVID-19 [14,15]. These findings underscore the importance of continued psychological monitoring and follow-up after hospital discharge. Early identification of patients with residual anxiety may allow timely referral for outpatient psychological support, potentially reducing the risk of long-term mental health sequelae following COVID-19 hospitalization.

Limitations

This study has certain limitations. The retrospective design restricts the ability to draw causal inferences, and the absence of a control group limits direct comparison with patients who did not receive psychological intervention. Additionally, follow-up was confined to the inpatient period, and longer-term psychological outcomes could not be assessed. Accordingly, the findings should be interpreted as descriptive of short-term changes in anxiety among hospitalized patients rather than as evidence of intervention efficacy.

## Conclusions

Anxiety is prevalent among patients hospitalized with mild COVID-19. Structured psychological intervention during hospitalization was associated with significant short-term improvement in anxiety severity in our cohort; however, causality cannot be definitively established due to the study design. Incorporating routine psychological screening and early non-pharmacological intervention may enhance inpatient care and improve patient well-being.
